# HTR4 gene structure and altered expression in the developing lung

**DOI:** 10.1186/1465-9921-14-77

**Published:** 2013-07-26

**Authors:** Emily Hodge, Carl P Nelson, Suzanne Miller, Charlotte K Billington, Ceri E Stewart, Caroline Swan, Anders Malarstig, Amanda P Henry, Catherine Gowland, Erik Melén, Ian P Hall, Ian Sayers

**Affiliations:** 1 Division of Respiratory Medicine, University of Nottingham, Queen’s Medical Centre, Nottingham NG7 2UH, UK; 2 Institute of Environmental Medicine, Karolinska Institutet and Sachs’ Children’s Hospital, Stockholm, Sweden; 3 Precision Medicine Unit, Pfizer Global Research and Development, Cambridge, UK

**Keywords:** 5-hydroxytryptamine, *HTR4*, 5-HT_4_R, Splice variant, Lung development, COPD, GPCR

## Abstract

**Background:**

Meta-analyses of genome-wide association studies (GWAS) have identified single nucleotide polymorphisms (SNPs) spanning the 5-hydroxytryptamine receptor 4 (5-HT_4_R) gene (*HTR4*) associated with lung function. The aims of this study were to i) investigate the expression profile of *HTR4* in adult and fetal lung tissue and cultured airway cells, ii) further define *HTR4* gene structure and iii) explore the potential functional implications of key SNPs using a bioinformatic approach.

**Methods:**

Following reverse transcription (RT)-PCR in human brain, 5′ rapid amplification of cDNA ends (5′ RACE) was used to examine the exonic structure of *HTR4* at the 5′ end. Quantitative (Q)-PCR was used to quantify *HTR4* mRNA expression in total RNA from cultured airway cells and whole lung tissue. Publically available gene microarray data on fetal samples of estimated gestational age 7–22 weeks were mined for *HTR4* expression. Immunohistochemistry (IHC; in adult and fetal lung tissue) and a radioligand binding assay (in cultured airway cells) were used to analyze 5­HT_4_R protein expression.

**Results:**

IHC in adult lung, irrespective of the presence of chronic obstructive pulmonary disease (COPD), suggested low level expression of 5-HT_4_R protein, which was most prominent in alveolar pneumocytes. There was evidence of differential 5-HT_4_R protein levels during gestation in fetal lung, which was also evident in gene expression microarray data. *HTR4* mRNA expression, assessed by Q-PCR, was <0.5% relative to brain in total adult lung tissue and in human airway smooth muscle (HASM) and bronchial epithelial cells (HBEC) derived from adult donors. Radioligand binding experiments also indicated that HBEC and HASM cells did not express a significant 5-HT_4_R population. 5′ RACE in brain identified a novel *N*-terminal variant, containing an extended *N-*terminal sequence. The functional significance of key *HTR4* SNPs was investigated using the encyclopedia of DNA elements consortium (ENCODE) dataset. These analyses identified multiple alterations in regulatory motifs for transcription factors implicated in lung development, including Foxp1.

**Conclusions:**

Taken together, these data suggest a role for *HTR4* in lung development, which may at least in part explain the genetic association with lung function.

## Background

5-Hydroxytryptamine (5-HT or serotonin) is a highly conserved monoamine, which is a major neurotransmitter in the CNS. However, 5-HT is also widely distributed throughout the periphery, with critical roles identified in cardiovascular physiology, gastrointestinal and endocrine function, the regulation of food intake and energy balance, as well as pulmonary physiology [1]. These myriad functions are performed through at least 15 distinct receptors, grouped into seven structurally and functionally-defined families (5-HT_1_ – 5-HT_7_ receptors, encoded by *HTR1-HTR7* genes) [2]. With the exception of the 5-HT_3_ receptor, which is a ligand-gated ion channel, these 5-HT receptors (5-HTRs) are members of the G protein-coupled receptor (GPCR) superfamily. Further functional diversity arises from alternative splicing (in the case of 5-HT_4_R and 5-HT_7_R), RNA editing (of the 5-HT_2C_ receptor) and both homo- and hetero-dimerization involving a variety of 5-HTRs [3,4].

Recent genome-wide association studies (GWAS) have identified an association between single nucleotide polymorphisms (SNPs) localised to a region encompassing the *HTR4* gene and lung function, assessed by forced expiratory volume in 1 second (FEV_1_) and the ratio of FEV_1_ to forced vital capacity (FVC) [5-7]. Subsequent studies have demonstrated an association between this locus and chronic obstructive pulmonary disease (COPD) [8] and airflow obstruction in smokers [9].

The complexity of the serotonin receptor family is exemplified by the *HTR4* sub-family. Encoded by a complex gene spanning ~200 kb on chromosome 5q33, *HTR4* has at least 10 human splice variants identified to date. Numerous studies in recombinant systems have demonstrated that alternative splicing of the *HTR4* gene can generate receptor species with distinct pharmacological and functional profiles, although all couple positively to adenylyl cyclase, leading to cyclic AMP generation (see [10] for review). The majority of these splice variants share a common primary sequence for the first 358 residues, only diverging at the *C*-terminus. The exception is 5-HT_4h_R, which possesses an additional 14 residues in the second extracellular loop of the receptor and has been found in combination with the 5-HT_4b_R *C*-terminal sequence [11].

*HTR4* is highly expressed in the central nervous system, particularly in limbic structures, where it has been implicated in learning and memory, depression, anxiety and feeding behaviour [12]. Peripherally, roles for 5-HT_4_R have been identified in the gastrointestinal tract, heart, vasculature, adrenal cortex and lower urinary tract [1,13]. Low levels of *HTR4* transcript have also been detected in lung [6,14,15] and in airway epithelial and smooth muscle cells [6,16,17].

Given the genetic association data and potential clinical significance of the *HTR4* gene in respiratory physiology and pathophysiology, we sought to i) investigate the expression profile of *HTR4* in both adult and fetal lung tissue and in lung tissue from individuals with COPD, ii) define the gene structure and iii) using the ENCODE dataset, investigate the potential functional mechanisms underlying select key *HTR4* SNPs associated with lung function. Our data demonstrate that in adult human lung tissue and isolated cells, including airway smooth muscle and bronchial epithelial cells, *HTR4* expression is very low at both protein and mRNA levels. Similar findings were observed in lung tissue isolated from COPD patients. Interestingly, we identify that 5-HT_4_R is differentially expressed across developmental stages, potentially suggesting a role for this receptor in lung development. Finally, we have identified a novel splice variant at the *N*-terminus and multiple potential regulatory mechanisms, which may underlie the observed *HTR4* SNP associations with lung function, including the alteration of transcription factor binding sites for factors linked to lung development.

## Methods

### Immunohistochemistry (IHC)

Three undiseased adult lung samples and three lung samples from individuals with clinically diagnosed COPD were collected from the Nottingham Health Science Biobank (Nottingham, UK) with the required ethical approval (08/H0407/1). Twelve fetal tissue samples were collected from the Human Developmental Biology Resource (Newcastle upon Tyne and London, UK, http://www.hdbr.org) at diverse stages of development, specifically 19, 21 and 23 days and 10, 12, 17 and 19 weeks post-conception. Samples were consented for in accordance with national banking procedures and the UK Human Tissue Act (2004). For all samples, 4 μm whole tissue sections on glass slides were de-paraffinized in Histo-clear (National Diagnostics, Dublin, Ireland) and hydrated using decreasing concentrations of ethanol. Antigen retrieval was performed in a steamer for 20 minutes in sodium citrate buffer (pH 6.0), followed by an endogenous peroxidise block for 5 minutes (Dako, Cambs, UK). Slides were incubated with either a rabbit polyclonal anti-5-HT_4_R antibody (1:500, ab60359, Abcam, Cambridge, UK) or treated with normal rabbit IgG as a matched isotype control (Invitrogen/Life Technologies, Paisley, UK) for 1 hour at room temperature. The Dako Chemate Envision Detection Kit (Dako) with DAB chromogen was used for detection. Sections were then counterstained with Mayer’s Haematoxylin (Sigma-Aldrich, Dorset, UK), dehydrated and a coverslip mounted using Vectamount (Vector Laboratories, Peterborough, UK). Human brain tissue was used as a positive control for 5-HT_4_R staining, whilst a negative control substituted the primary antibody with antibody diluent. Results were visualized using an Olympus BX14 light microscope.

### Cell culture and transfection

All cells were maintained at 37°C in 5% CO_2_ in a humidified incubator. Human airway smooth muscle (HASM) cells were isolated from the healthy bronchial tissue of individuals without previous asthma history undergoing surgery, and cultured as previously described [18]. Approval was given by the Nottingham Local Ethical Research Committee (EC00/165). Undifferentiated human bronchial epithelial cells (HBEC) (Lonza/Biologics, Slough, UK) were maintained in culture and differentiated, where relevant, as previously described [19]. *HTR4* mRNA expression analysis was carried out in five HASM (passage 3–5) and four HBEC (passage 3) donors, while radioligand binding experiments were performed on at least two distinct HASM and HBEC donors. CHO-K1 cells and the human bronchial epithelial cell line BEAS2B-R1 [20] (provided by Dr. Ray Penn, University of Maryland, Baltimore, USA) were cultured in Dulbecco’s Modified Eagle’s Medium supplemented with 10% fetal calf serum (FCS; Sigma-Aldrich). CHO-K1 cells were transfected (where appropriate) in 24-well plates with a pcDNA3-HTR4a plasmid (1 μg/well) using FugeneHD transfection reagent (Promega, Southampton, UK), according to manufacturer’s instructions. The pcDNA3-HTR4a plasmid was constructed by amplifying the protein-coding region of the *HTR4a* transcript from total brain RNA (Ambion/Life Technologies) and inserting this into the EcoRI site of the pcDNA3 vector. The insert in the resulting plasmid was sequence-verified as for RT-PCR products (see below).

### Reverse-Transcription PCR (RT-PCR)

RT-PCR used cDNA synthesised from total RNA using the Superscript First-Strand Synthesis System for RT-PCR (Invitrogen/Life Technologies), extracted from cultured cells or commercially obtained (peripheral blood mononuclear cells (PBMC; 3H Biomedical AB, Uppsala, Sweden), total lung and brain tissue (both from Ambion/Life Technologies)). Initial *HTR4* expression analysis by RT-PCR used a forward primer binding in exon 1 (primer 1F, 5′-CAGCAGAAGCTCGGCTCAG-3′) with reverse primers binding either in exon 9 (primer 9R, 5′-CTCTCATGGCTGTCTTCTGG-3′) or exon 13 (primer 13R, 5′-CAATCAGAAGCATGATTCCAG-3′), with the following cycling parameters: 35 cycles of 94°C for 1.5 minutes, 60°C for 1.5 minutes, 72°C for 1.5 minutes, followed by 72°C for 10 minutes. Expression analysis of the novel *HTR4* variant transcript used a forward primer binding in the novel exon (primer novel_F, 5′-GAATGGAGAGATCCAGATGG-3′) and primer 9R, with the following cycling protocol: 94°C for 2 minutes, then 40 cycles of 94°C for 30 seconds, 55°C for 30 seconds, 68°C for 1.5 minutes, followed by 68°C for 5 minutes. Amplicons were extracted from agarose gels using the QIAquick Gel Extraction Kit (Qiagen, Crawley, UK) and sequence verified using the BigDye Terminator v3.1 Cycle Sequencing Kit in conjunction with an ABI PRISM 310 Genetic Analyzer (Applied Biosystems/Life Technologies).

### Quantitative PCR (Q-PCR)

Expression analysis of *HTR4* was carried out in total RNA from cultured cells or commercially available total lung and brain tissue (Ambion/Life Technologies) using TaqMan methodology for real-time Q-PCR (Applied Biosystems/Life Technologies) as previously described [19]. In every sample, Q-PCR was carried out for *HTR4* (forward primer 5′-TCTCTTGCTTTTGCGGATCT-3′, reverse primer 5′-GCAGAGGGGTCATCTTGTTC-3′, probe 5′-CCCTTTGGTGCCATTGAGCTGGTTC-3′) and Human TFRC (CD71, transferrin receptor) Endogenous Control (Applied Biosystems/Life Technologies). Relative *HTR4* expression was calculated using the 2^-ΔΔCt^ method [21], corrected using the endogenous control (TFRC) and displayed relative to expression in total brain tissue.

### Affymetrix U133 Plus 2 array data for human fetal lung

Publically available data [22,23] were utilized to see whether *HTR4* was differentially expressed during normal human lung development. Previously, human fetal lung tissues were obtained from National Institute of Child Health and Human Development tissue databases and microarray profiled to investigate the expression spanning different gestational ages. RNA samples from 38 subjects (estimated gestational age 7–22 weeks or 53–154 days post conception) *i.e.* Pseudoglandular (gestational age, 7–16 weeks) and Canalicular (17–26 weeks) stages of development were included within the dataset. These data are available at NCBI Gene Expression Omnibus (GEO, http://www.ncbi.nlm.nih.gov/geo), GSE14334. The dataset was mined for *HTR4* expression using Affymetrix probes; 216939_s_at, 207577_at and 207578_s_at.

### Radioligand binding

CHO-K1 cells were plated into 24-well plates and grown for 24 hours in DMEM containing 10% FCS prior to transfection. BEAS2B-R1, HBEC and HASM cells were plated into 24-well plates 24–48 hours prior to experimentation. Experiments were performed 48 hours post-transfection in HEPES-buffered saline (HBS: 20 mM HEPES, 150 mM NaCl, 4.2 mM KCl, 0.9 mM CaCl_2_, 0.5 mM MgCl_2_, 0.1% glucose, and 0.1% bovine serum albumin), as previously reported [24]. The reaction mixture (250 μl) containing saturating concentrations of [^3^H]-GR113808 (approx. 1 nM, *K*_d_ = 0.30 nM; specific activity, 83 Ci/mmol) was added to the cells and incubated for 2 hours at 4°C. Non-specific binding was defined in the presence of 5-HT (10 μM). Incubation was terminated by aspiration, followed by washing the cells with ice-cold HBS (2 × 1 ml). Cells were lysed in 0.1 M NaOH, then 10 ml of scintillation liquid (UltimaGold XR scintillation cocktail; Perkin Elmer, Bucks, UK) was added and radioactivity was measured by scintillation counting. Protein concentration was determined using a Bio-Rad protein assay (Hercules, California, USA), allowing receptor densities (in fmol/mg protein) to be determined.

### 5′ Rapid amplification of cDNA Ends (5′ RACE)

Total RNA was extracted from cultured cells using the RNeasy Mini Kit (Qiagen) or obtained from commercial sources. RACE-ready cDNA was synthesised using total RNA from HASM, undifferentiated and differentiated HBEC, PBMC (3H Biomedical AB) and total lung and brain tissue (Ambion/Life Technologies) using the GeneRacer Kit (Invitrogen/Life Technologies). 5′ RACE used GeneRacer primers as well as *HTR4*-specific nested primers for amplification. PCR products generated were then cloned using the pGEM-T Easy Vector System (Promega) to identify exon structure at the 5′ end of the gene. RACE clones were sequenced using the BigDye Terminator v3.1 Cycle Sequencing Kit in conjunction with an ABI PRISM 310 Genetic Analyzer (Applied Biosystems/Life Technologies). Sequences were aligned with the ‘Human Genomic + Transcript’ database using the Basic Local Alignment Search Tool (BLAST) provided by the National Center for Biotechnology Information (NCBI, http://www.ncbi.nlm.nih.gov/). Protein structures were predicted from 5′ RACE data using online tools: the TMHMM Server v. 2.0 [25] and TOPO2, provided by the Sequence Analysis & Consulting Service at UCSF (http://www.sacs.ucsf.edu/cgi-bin/open-topo2.py/).

### Bioinformatic analysis

The *HTR4* gene was annotated with RNA sequencing, H3K27Ac histone marks, DNase I hypersensitivity, transcription factor binding (all derived from the UCSC encyclopedia of DNA elements consortium (ENCODE) database [26]), CpG island and placental mammal conservation tracks using the UCSC genome browser (http://genome.ucsc.edu/) on the Human Feb 2009 (GRCh37/hg19) assembly [27,28]. The predicted effects of polymorphisms (in the HapMap CEU cohort, as of December 2012) in linkage disequilibrium (LD) (r^2^>0.80) with the sentinel *HTR4* SNP (rs3995090) identified in the SpiroMeta lung function GWAS [6] were examined using the HaploReg database (http://www.broadinstitute.org/mammals/haploreg/) [29].

## Results

### 5-HT_4_R is expressed at low levels in adult human lung tissue

**Figure 1:**
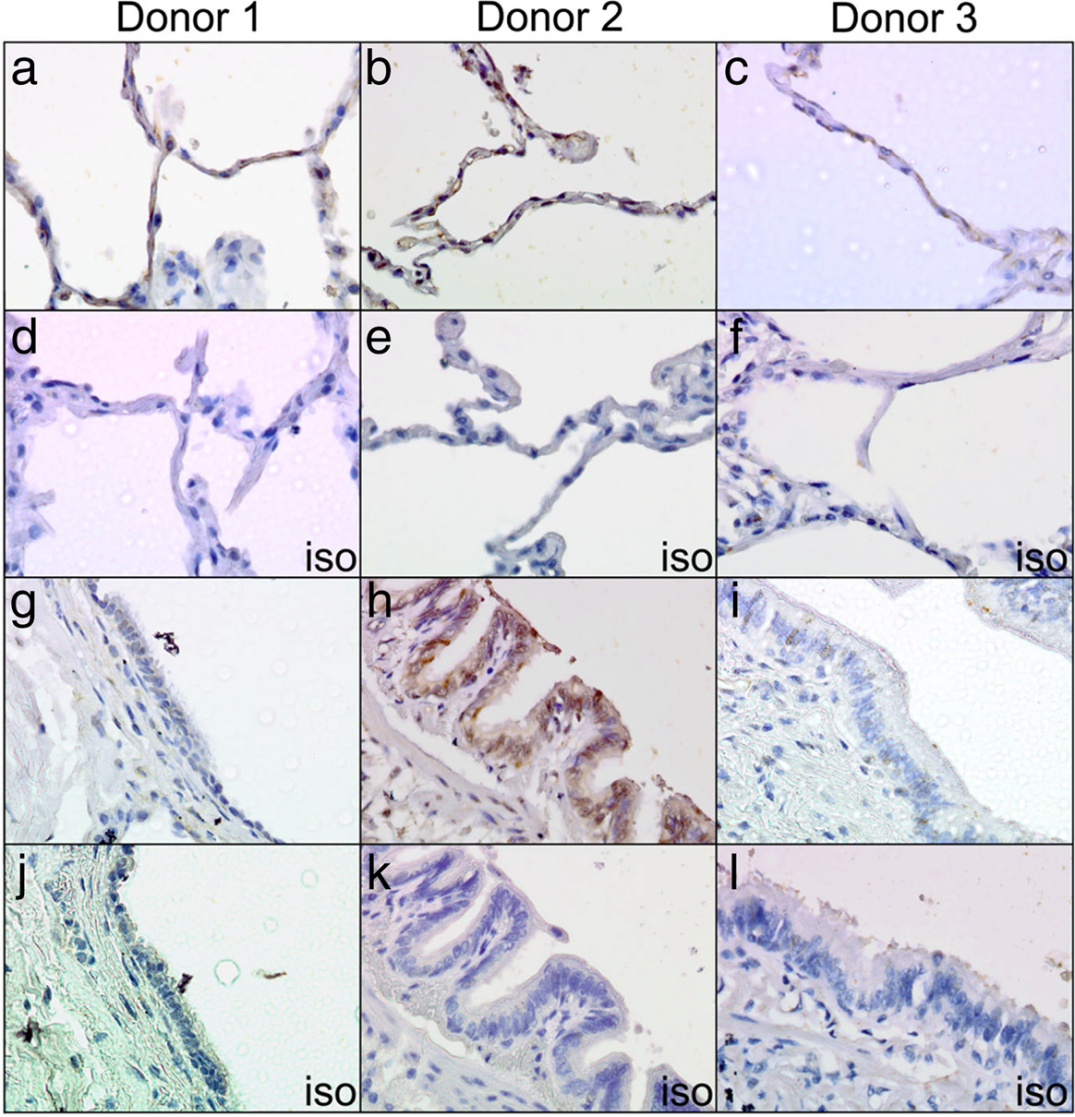
**Immunohistochemistry of 5-HT**_**4**_**R in normal adult human lung tissue.** Low cytoplasmic and membranous expression of 5-HT_4_R was found in the pneumocytes of the alveoli in all three normal lung samples **(a**-**c)**. The epithelium showed variable staining; donor 1 had very weak cytoplasmic staining **(g)**, donor 2 had strong cytoplasmic and nuclear staining of >50% of nuclei **(h)**, whilst donor 3 had weak staining in <20% of epithelial cells **(i)**. All isotype controls were negative **(d**-**f** and **j**-**l)**. X40 magnification.

Immunohistochemistry performed with an anti-5-HT_4_R antibody in normal lung tissue from three donors identified specific staining for 5-HT_4_R in alveolar pneumocytes (Figure 1a-c). The staining in these cells was both cytoplasmic and membranous, which is consistent with the anticipated sub-cellular expression profile for a GPCR. Some specific 5-HT_4_R staining was also detected in bronchial epithelial cells, but substantial variation between donors was observed in this location. While strong staining was observed in the epithelial cells of donor 2 (Figure 1h), only weak staining was observed in the other 2 donors (Figure 1g and 1i). In addition, the strong staining in donor 2 was cytoplasmic and nuclear, with little apparent staining at the plasma membrane (Figure 1h). No staining was observed in any of the isotype controls (Figure 1d-f and 1j-l).

### 5-HT_4_R is expressed at low levels in adult human lung tissue from individuals with COPD

**Figure 2:**
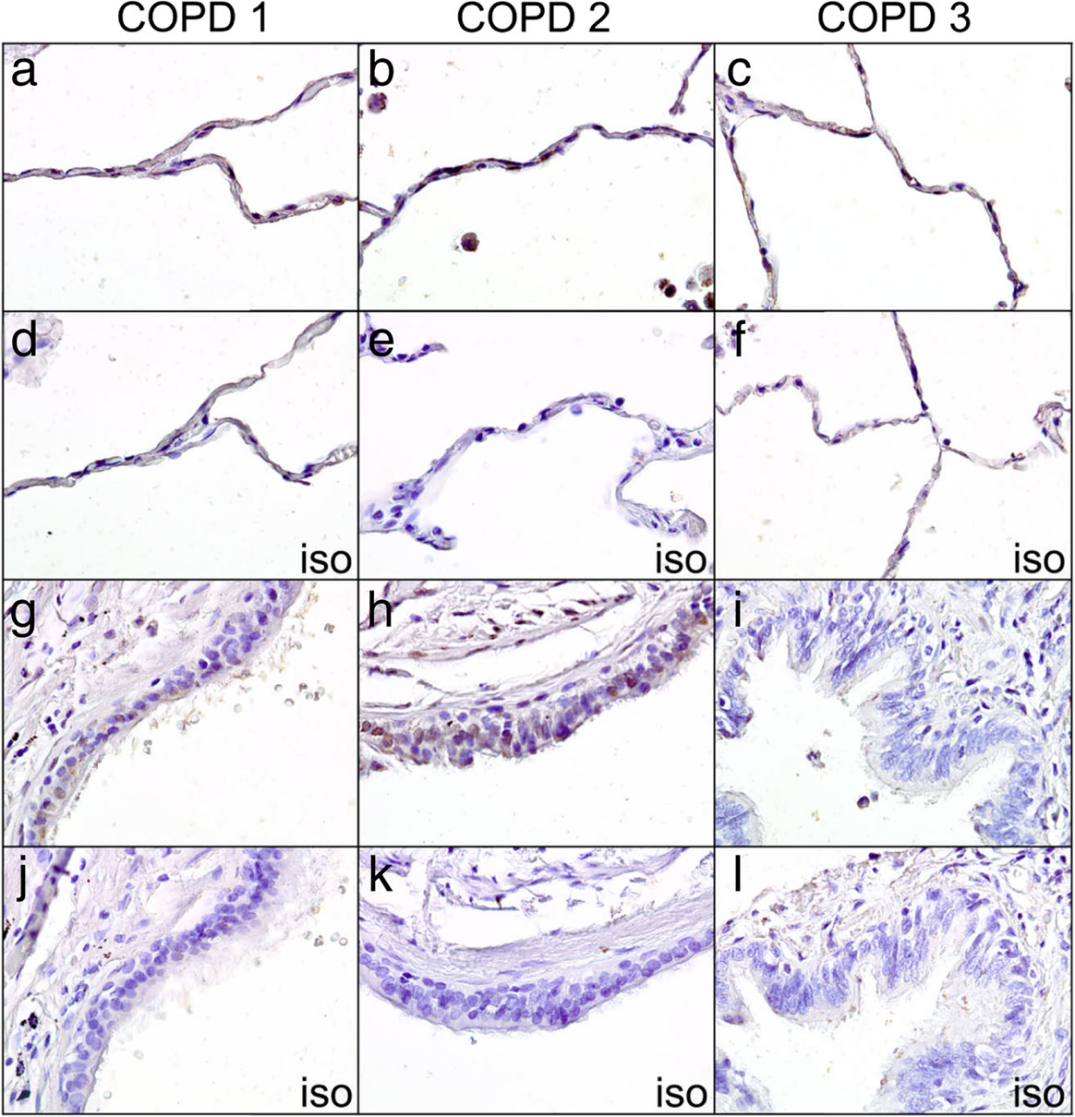
**Immunohistochemistry of 5-HT**_**4**_**R in COPD lung tissue.** Weak 5-HT_4_R staining was found in the nuclei and cytoplasm of pneumoctyes in the alveolar regions of all COPD samples **(a**-**c)**. In the bronchial epithelium COPD1 had weak nuclear staining **(g)**, COPD2 had moderate nuclear and cytoplasmic staining **(h)** whilst COPD3 was negative for the 5-HT_4_R protein **(i)**. All isotype controls were negative **(d**-**f** and **j**-**l)**. X40 magnification.

5-HT_4_R protein expression was analyzed by IHC in three COPD donors. Donor 1 (COPD1) was a 61 year old female, heavy smoker with moderate COPD; donor 2 (COPD2) was a 55 year old male, heavy smoker with severe COPD; donor 3 (COPD3) was a 48 year old female, heavy smoker with severe COPD. Weak 5-HT_4_R immunopositivity was found in the nuclei and cytoplasm of pneumoctyes in the alveolar regions of all three COPD samples (Figure 2a-c). The epithelium showed variable staining between donors: COPD1 had weak nuclear staining (Figure 2g), COPD2 had moderate nuclear and cytoplasmic staining (Figure 2h) whilst COPD3 was negative for the 5-HT_4_R protein (Figure 2i). All isotype controls were negative (Figure 2d-f and 2j-l).

### 5-HT_4_R is differentially expressed during fetal development at the protein and mRNA level

**Figure 3:**
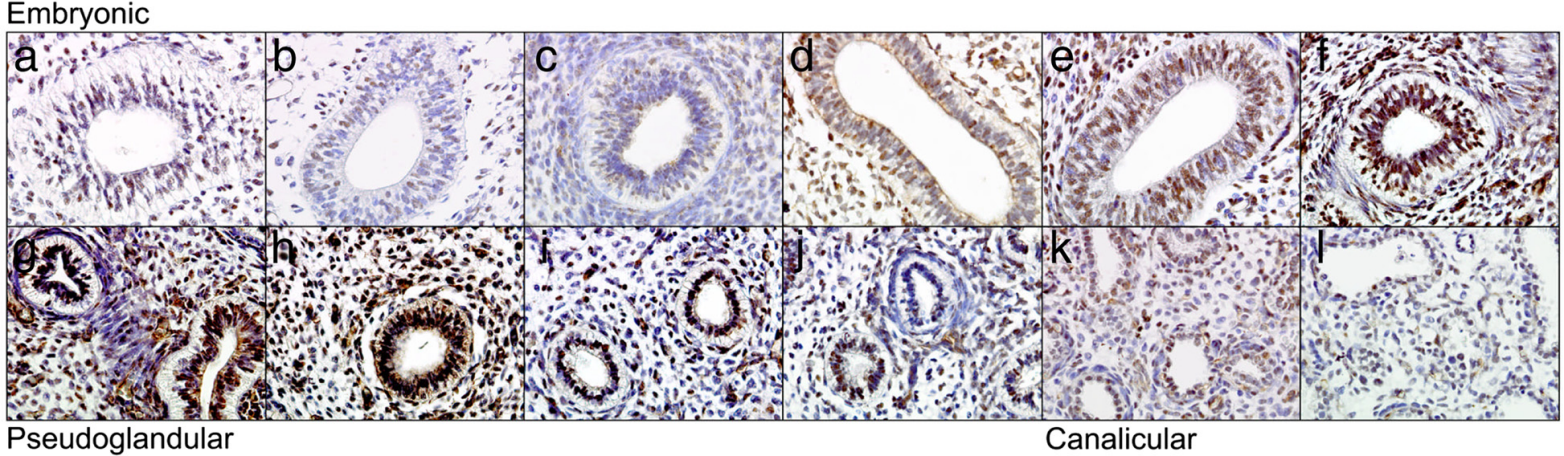
**Immunohistochemistry of 5-HT**_**4**_**R in fetal lung tissue.** Weak staining is apparent in early Embryonic stages which increases in intensity with age leading to strong staining in the Pseudoglandular and moderate staining in the Canalicular stages. **a**-**l** represent age ordered samples of 19 days **(a**, **b)**, 21 days **(c**, **d)**, 23 days **(e**, **f)**, 10 weeks **(g**, **h)**, 12 weeks **(i**, **j)**, 17 weeks **(k)** and 19 weeks **(l)**. All isotype controls were negative (data not shown). X40 magnification.

Fetal tissue spanning ages 19 days-19 weeks was investigated using IHC (Figure 3). These data demonstrated a trend towards an increase in 5-HT_4_R protein expression spanning Embryonic (Figure 3a-f) to Pseudoglandular (Figure 3g-j) stages and a potential decrease in the Canalicular (Figure 3k-l) stage of development. Staining was predominantly nuclear and widespread across the airway epithelium. The gene array data demonstrated that there was a significant increase in expression levels with age during the Pseudoglandular (gestational age, 7–16 weeks) and Canalicular (17–26 weeks) stages of development, with one of the Affymetrix probes (207577_at) surviving correction for false discovery rate (Table 1). These data suggest that an increase in 5-HT_4_R expression is a feature of lung development with a lower level of protein expression in subsequent stages, potentially reflecting the levels observed in the adult lung.

**Table 1:** **Fetal lung gene array data for *HTR4 *expression during Pseudoglandular and Canalicular stages of lung development**

**Probe ID**	**AveExpr**	**t**	**P.value**	**Adj.p.val**	**Beta-coefficient**
216939_s_at	3.2999	0.9672	0.3393	0.5156	0.0009
207577_at	3.6980	3.3242	0.0019	0.0121	0.0024
207578_s_at	7.0472	0.3855	0.7020	0.8160	0.0008

*AveExpr*, average expression between all samples; *t*, t-statistic describing differential expression; *P.Value*, Unadjusted p value; *adj.P.Val*, Adjusted p value controlling for false discovery rate; Beta-coefficient= log-odds ratio (corresponding to the mean change in gene expression per day during the studied period, 7–22 weeks of gestational age).

### Analysis of *HTR4* gene structure in brain and *HTR4* expression profiling in airway cells by Q-PCR and radioligand binding

**Figure 4:**
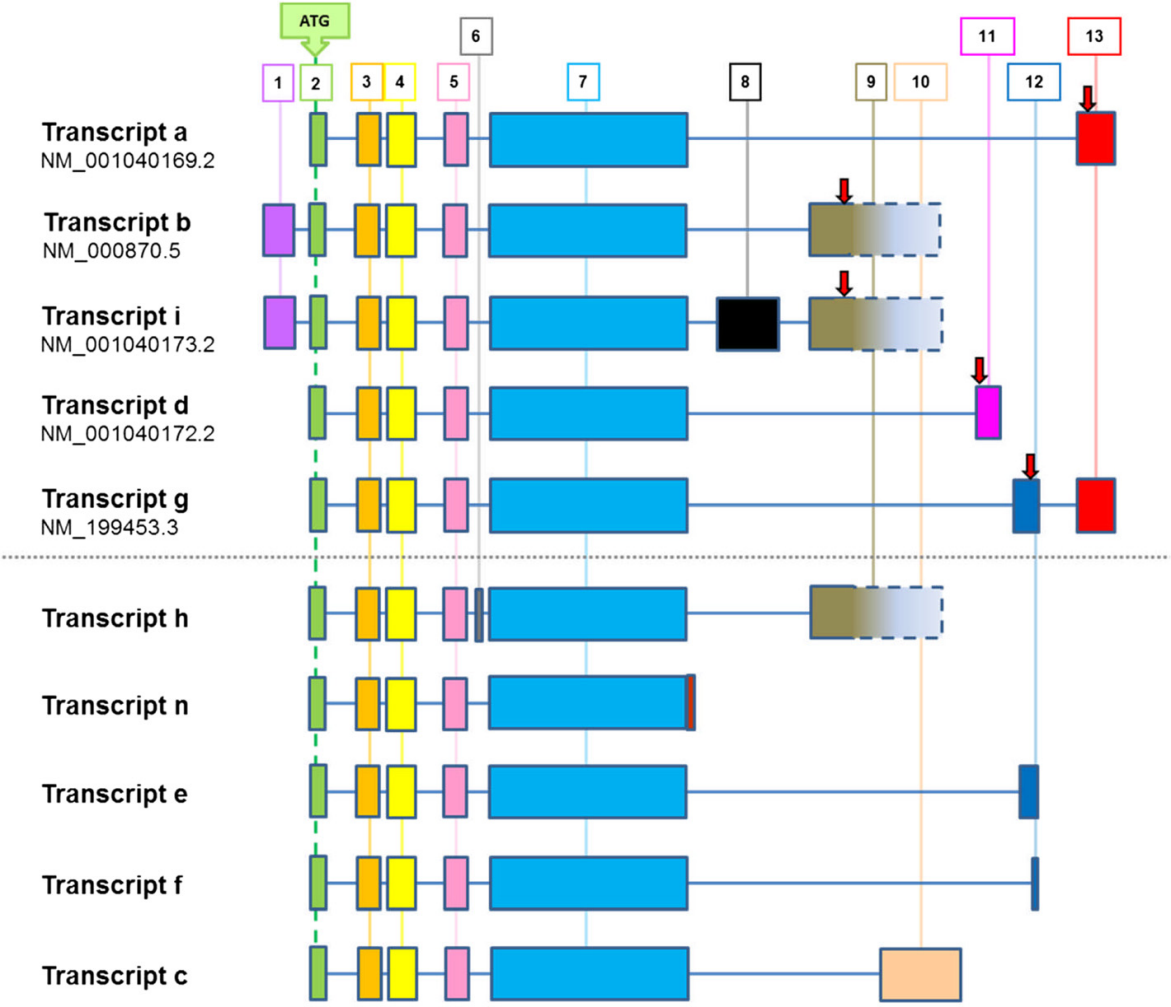
**Known *HTR4 *transcript variants.** Transcripts a, b, i, d and g are currently reported by NCBI (December 2012); translational stop positions are indicated by red arrows. Transcripts h, n, e, f and c have been described in the literature [11,15,30-32].

**Figure 5:**
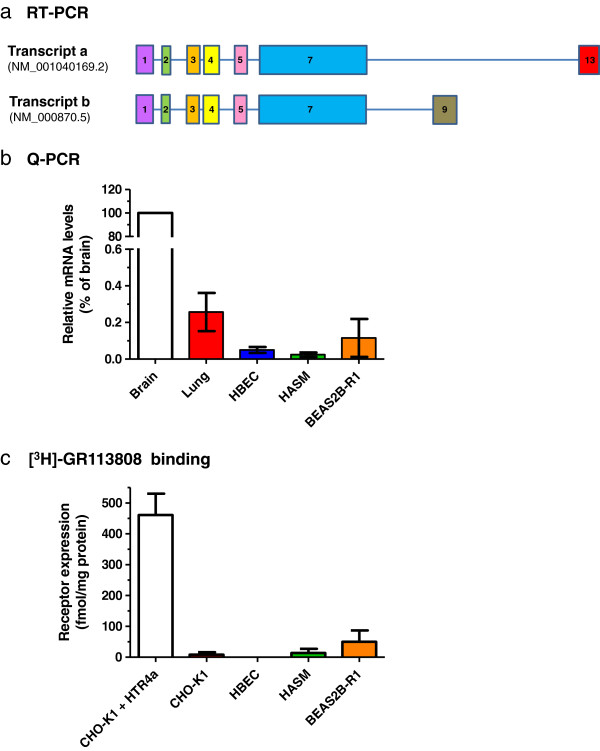
**Expression profiling of *HTR4 *in airway cells.** RT-PCR and sequencing confirmed expression of *HTR4* transcripts a (NM_001040169.2) and b (NM_000870.5) in total brain tissue **(a)**. Sequence encoding exon 1 was found in both transcripts, which contradicts information provided by NCBI for transcript a, however transcripts a and b were differentiated by the presence of exon 13 or 9 respectively. Q-PCR analysis **(b)** indicated highest expression in total brain tissue, but ≤0.26% relative to this in total lung tissue, HBEC, HASM and the BEAS2B-R1 cell line. Error bars indicate SEM (n ≥3). Radioligand binding experiments **(c)**, using a saturating concentration of [^3^H]-GR113808 (approx. 1 nM), defined 5-HT_4_R expression in CHO-K1 cells transiently transfected with pcDNA3-HTR4a, un-transfected CHO-K1 cells, HBEC, HASM and BEAS2B-R1 cells. Error bars indicate SEM (n ≥3). While the pcDNA3-HTR4a-transfected CHO-K1 cells expressed a substantial 5-HT_4_R population (approx. 460 fmol/mg protein), no other cell type expressed a significant receptor population (all ≤50 fmol/mg protein).

Previously described gene arrangements for *HTR4* are summarized in Figure 4. Transcripts a, b, i, d and g are currently reported by NCBI (December 2012); transcripts h, n, e, f and c have been described in the literature [10]. These data illustrate the complex splicing that exists at this gene locus, including multiple functionally relevant *C-*terminal variants, leading to alterations in cyclic AMP signalling responses [10]. Analysis by RT-PCR indicated expression of mRNA encoding *HTR4* in total brain tissue, but failed to detect expression in HASM, undifferentiated HBEC, PBMC or total lung tissue (data not shown). Targeted sequencing of PCR products derived from brain cDNA confirmed the presence of both variants a (accession NM_001040169.2) and b (accession NM_000870.5), distinguished by the presence of exons 9 and 13 respectively. Additionally, exon 1 was present in variant a, although this contradicts data currently reported by NCBI (Figure 5a). Q-PCR data indicated very low levels of mean *HTR4* mRNA expression in airway cells and tissues when compared to total brain tissue: total lung (0.26%), undifferentiated HBEC (0.05%), HASM (0.02%) and BEAS2B-R1 (0.16%) (Figure 5b).

In addition, radioligand binding (using the highly 5-HT_4_R-selective ligand [^3^H]-GR113808 [24]) failed to detect any significant 5-HT_4_R expression in undifferentiated HBEC (none detected; n = 3), HASM (14.1 ± 13.2 fmol/mg protein; n = 4) or BEAS2B-R1 (50.1 ± 36.6 fmol/mg protein; n = 3) cells (Figure 5c), in good agreement with the Q-PCR data in these cell types. In contrast, a substantial 5-HT_4_R population could be detected in CHO-K1 cells transiently transfected with pcDNA3-HTR4a (460.7 ± 69.3 fmol/mg protein; n = 4), but not in un-transfected CHO-K1 cells (8.3 ± 7.8 fmol/mg protein; n = 4). These data suggest that there is limited 5-HT_4_R expression in cultured structural cells of the adult lung, in agreement with the low levels of IHC staining for 5-HT_4_R in adult lung tissue.

**Figure 6:**
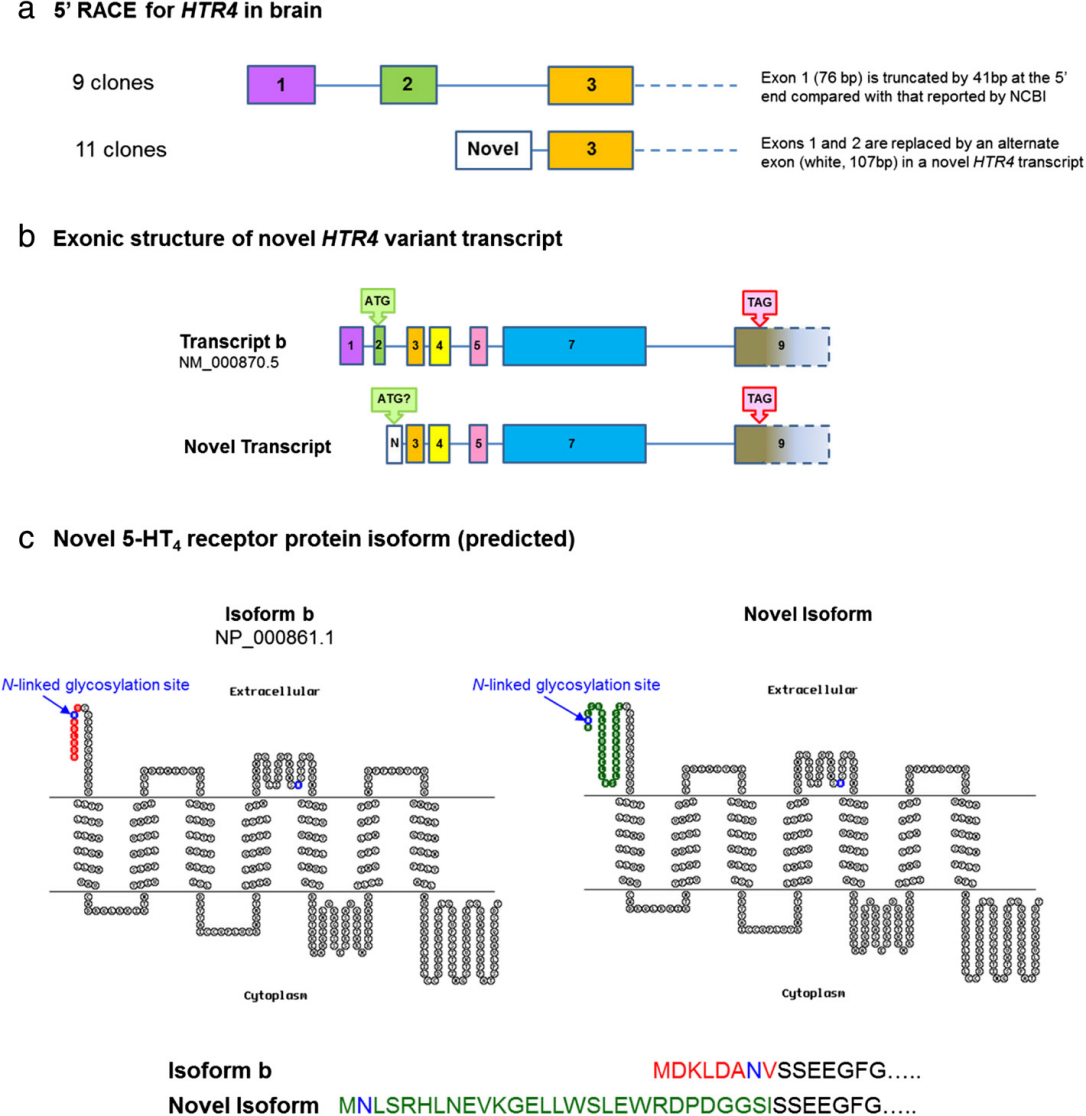
**Analysis of *HTR4 *gene structure and identification of a novel transcript variant in brain.** 5′ RACE in cDNA derived from total brain tissue indicated expression of transcripts possessing exons 1-3, and also a novel transcript in which exons 1 and 2 are replaced by an alternate exon **(a)**. The remaining sequence of the novel transcript aligns with the equivalent region of transcript b **(b)**. The predicted protein structure of this novel variant has an elongated *N*-terminus and affects the position of an *N*-linked glycosylation site in this region **(c)**.

5′ RACE showed variation of *HTR4* transcripts in cDNA derived from total brain tissue only; it was not possible to amplify *HTR4* transcripts from total lung tissue, HASM, HBEC (undifferentiated and differentiated) and PBMC due to the extremely low abundance. Approximately half of the analyzed clones derived from brain cDNA showed the presence of exons 1–3, although exon 1 was truncated by 41bp at the 5′ end compared to the sequence reported by NCBI (Figure 6a). The remaining clones possessed a novel exon of 107bp, in place of exons 1 and 2. Two consecutive RT-PCR procedures in cDNA derived from total brain, *i.e.* using the amplicon from the first as a template in a second, and confirmation by sequencing showed that the exonic structure of the novel exon-containing variant was comparable to transcript b, since sequence alignment was of good quality for consecutive exons 3, 4, 5, 7 and 9 (Figure 6b). However, consecutive RT-PCR procedures failed to detect this novel transcript in total lung tissue or differentiated HBEC (data not shown). Using BLAST, we found that the sequence of the novel exon aligns with a region on the same genomic contig as the *HTR4* gene (accession NG_029052.1). Using the same translational stop codon as that in transcript b, the novel transcript variant has an in-frame translational start codon (ATG) within this novel exon. The predicted protein structure of the novel transcript has an extended *N*-terminus (Figure 6c). Furthermore, the *N*-linked glycosylation site in the *N*-terminal region of transcript b is replaced in the novel transcript by an alternate site further upstream.

### Analysis of HTR4 gene regulation using the ENCODE dataset

**Figure 7:**
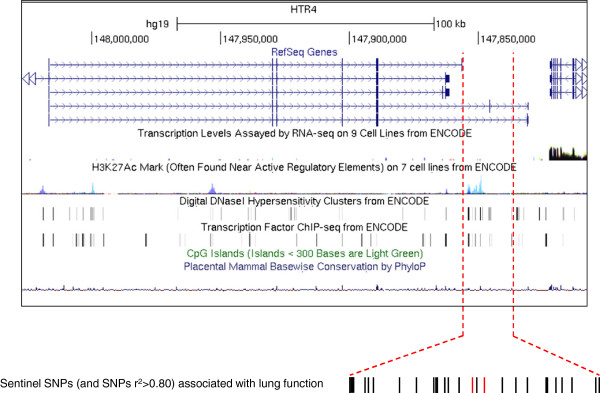
**Regulatory motifs and lung function-associated single nucleotide polymorphisms (SNPs) within the *HTR4 *gene.***HTR4* annotated with RNA sequencing, H3K27Ac histone marks, DNase I hypersensitivity, transcription factor binding, CpG island and placental mammal conservation tracks (UCSC genome browser (http://genome.ucsc.edu/)) on the Human Feb 2009 (GRCh37/hg19) assembly. For the H3K27Ac histone marks and RNA sequence tracks, peak height is proportional to signal amplitude, with colours representing data sets in different cell backgrounds (pale blue H3K27Ac histone trace = human umbilical vein endothelial cell (HUVEC); blue/grey = K562 erythroleukemia cells). For the DNase I hypersensitivity and transcription factor binding tracks, a grey band indicates the extent of the hypersensitive region and the darkness of the band is proportional to the maximum signal strength observed in any cell line. The positions of *HTR4* variants in LD (r^2^>0.80) with the sentinel SNP (rs3995090) identified in the SpiroMeta lung function GWAS [6] are also shown, with the top 2 ranking SNPs (rs3995090 and rs6889822) indicated in red.

Annotation of the region containing the *HTR4* gene (shown in Figure 7) illustrates the regulatory elements contained within this region, as predicted by the ENCODE database. There are no obvious CpG islands in this region, however substantial DNase I hypersensitivity clusters, transcription factor binding sites (identified by ChIP-seq) and H3K27Ac marks (at least in HUVEC and K562 erythroleukemia cells) are identified within the 3′ end of the *HTR4* gene. These regulatory features coincide with the region containing the variants in linkage disequlibrium (LD) (r^2^>0.80) with the sentinel SNPs (rs3995090 and rs6889822) identified in the SpiroMeta lung function GWAS [6], as indicated in Figure 7. Less pronounced histone modification peaks, in addition to clusters of transcription factor binding and DNase I hypersensitivity sites are found towards the 5′ end of the *HTR4* gene. Using the HaploReg database focussed to one of the sentinel SNPs (rs3995090) identified in the SpiroMeta lung function GWAS [6], we found that this SNP was in LD (r^2^>0.80) with an additional 28 SNPs in the 1000 Genomes dataset, which included the second sentinel SNP rs6889822 (r^2^>0.96) (Additional file 1: Table S1). We identified a wide range of alterations associated with these SNPs in: i) enhancer histone marks, ii) DNase I hypersensitivity sites, iii) proteins bound and iv) regulatory motifs. Of note, three of the SNPs (rs7733088, rs4705259 and 5:147836450) in LD (r^2^>0.80) with sentinel SNP rs3995090 significantly alter *Foxp1* binding motifs within the *HTR4* gene (Additional file 1: Table S1). The Fox family of transcription factors are key regulators of lung development.

## Discussion

The translation of GWAS findings to human biology remains a challenge. In the current study we have begun to interpret our recent finding that SNPs spanning the *HTR4* gene are associated with FEV_1_ in the general population [6]. To this end we have extensively expression profiled the 5-HT_4_R protein and *HTR4* mRNA in human lung tissue and cells. We identified that 5-HT_4_R protein is only weakly expressed in the adult human lung, irrespective of the presence of COPD. In agreement, we identified very low levels of *HTR4* mRNA and were unable to detect a significant 5-HT_4_R population in cultured airway structural cells, including bronchial epithelial and airway smooth muscle cells. Interestingly, when we investigated 5-HT_4_R in human fetal lung tissue we identified a robust level of protein expression that appeared to be differentially regulated through lung development. In agreement, gene expression array data also showed differential *HTR4* mRNA lung expression levels across gestational stages. These data are novel and potentially suggest a role for *HTR4* in lung development. We also identified a novel *N*-terminal splice variant in brain with potential implications for 5-HT_4_R function. Finally we investigated the functional relevance of key genome-wide significant SNPs and identified multiple potential functional mechanisms *e.g.* through the alteration of developmental transcription factor binding motifs.

The very low level of *HTR4* mRNA detected in lung in the present study is consistent with a number of previous studies. For instance, Bach *et al.*[14] used RT-PCR to screen a variety of tissues for the expression of *HTR4a* and *HTR4b* and successfully detected both in a number of tissues (*e.g.* brain and colon). However, they only showed “barely detectable” levels of *HTR4b* (and were unable to detect *HTR4a* at all) in the lung. Medhurst *et al.*[33] employed a Q-PCR approach using a pan *HTR4* PCR assay and detected only very low levels of *HTR4* in lung. In contrast, Brattelid *et al.*[15] were able to detect both *HTR4a* and *HTR4b* in lung by RT-PCR, while Q-PCR identified *HTR4a*, *HTR4b* and *HTR4g* in the same tissue. Specifically in airway smooth muscle cells, a gene microarray study identified *HTR4g* as the only significant *HTR4* species [17]. Together with our findings in control and COPD subjects, the consensus from the literature would suggest that *HTR4* is expressed in adult lung, albeit at very low levels.

The majority of the literature is limited to whole lung *HTR4* expression so, in addition to human lung tissue, we chose to focus on two specific, functionally and clinically relevant primary airway cell types (cultured human bronchial epithelial cells (HBEC) and airway smooth muscle cells (HASM)), as well as the human bronchial epithelial cell line BEAS2B-R1, to investigate whether *HTR4* was enriched within a specific lung cell type. We observed similar (or even lower) levels of *HTR4* mRNA in these individual cell types as in whole lung. We also failed to detect a significant level of expression at the protein level (using [^3^H]-GR113808 radioligand binding) in these cells, despite successfully detecting robust 5-HT_4_R expression in pcDNA3-HTR4a-transfected CHO-K1 cells. Since [^3^H]-GR113808 has also previously been used to measure relatively low levels of endogenous 5-HT_4_R expression (60–223 fmol/mg protein) in human brain regions [34], our findings suggest that there is little enrichment of 5-HT_4_R in bronchial epithelial and airway smooth muscle cells. While the radioligand and IHC approaches to determine 5-HT_4_R protein levels in airway structural cells are in broad agreement, we cannot exclude that the lack of 5-HT_4_R protein expression observed in cultured cells is due to loss of receptor expression during passage. Bayer *et al.*[16] have previously identified *HTR4* expression in BEAS2B and A549 airway epithelial cell lines and in primary type II alveolar pneumocytes by RT-PCR, providing evidence for expression of *HTR4* in airway epithelial cells (albeit cell lines). Our immunohistochemical data indicate the strongest 5-HT_4_R protein expression in alveolar pneumocytes, with only weak and variable expression in bronchial epithelial cells. Taken together, these findings suggest that the main site of 5-HT_4_R expression in adult lung may be in alveolar pneumocytes.

In contrast to the adult human lung analyses, we observed clear, robust staining for 5-HT_4_R in the fetal lung tissue using immunohistochemistry. The predominantly nuclear localization of 5-HT_4_R in lung tissue suggests that the protein could have distinct roles during development, possibly beyond the scope of a classical plasma membrane-localized GPCR. Interestingly, there is a growing body of evidence for the nuclear localization and function of endogenous GPCRs in a variety of systems (see [35] for review). Notwithstanding the predominantly nuclear localization, our qualitative data identified a differential expression of 5-HT_4_R with gestational age, with the intensity of staining being particularly prominent in the Pseudoglandular stage of lung development. In agreement, using more quantitative mRNA data, differential expression through development was observed, with elevated *HTR4* expression across the Pseudoglandular (7–16 weeks) and Canalicular (17–26 weeks) stages. Although both immunohistochemical protein and expression array mRNA data indicate that there is differential *HTR4* gene expression during lung development, the timing of the increase in expression differs between mRNA (higher expression during the Canalicular stage) and protein (peak expression in the Pseudoglandular stage). For the gene expression microarray analysis, 26 samples in the Pseudoglandular stage (specifically 53 – 110 days post-conception) and 12 samples in the Canalicular stage (specifically 113 – 154 days post-conception) contributed to these analyses, whereas tissue used for immunohistochemistry included 4 samples at the Pseudoglandular stage (70 – 94 days post-conception) and only 2 samples at the Canalicular stage (specifically 119 and 133 days post-conception). It is, therefore, possible that the differences in sample sizes and/or sample cohorts used in the two analyses could underlie this discrepancy.

Nonetheless, these lung-specific data are novel and suggest *HTR4* is likely to be of relevance to lung development. Of interest, differential 5-HT_4_R protein expression during human hypothalamus development has been described, with the appearance of 5-HT_4_R being associated with the later stages (31–32 weeks) of development [36]. Similarly a role for 5-HT_4_R and other serotonin receptors has also been shown in the prefrontal cortex during post-natal development, again with elevated 5-HT_4_R expression reported during the later stages [37]. Taken together these data suggest a role for *HTR4* in the development of multiple human tissues.

The low level of *HTR4* mRNA detected in adult lung precluded a thorough investigation of the splice variant expression profile in human airways. However, there is extensive evidence for functionally relevant *HTR4* splicing, in particular at the *C*-terminus [10]. In brain, we were able to successfully perform 5′ RACE, which indicated significant expression of a previously un-reported *HTR4* splice variant, arising from the replacement of exons 1 and 2 by a novel exon. The resulting variant is predicted to possess an extended *N*-terminus (14 amino acids longer than the other 5-HT_4_R isoforms) and a distinct *N-*glycosylation site (at position 2 in the novel variant, as opposed to position 7 in all other reported human 5-HT_4_R variants). Interestingly, although our findings represent the first example of variation at the *N*-terminus of the human 5-HT_4_R, several transcripts encoding different *N*-termini have been identified in mouse brain [38]. Sequence predictions based upon these novel transcripts indicated changes in *N*-terminal length and in some cases altered phosphorylation, acetylation and glycosylation sites [38], highlighting parallels with our own findings in human brain. *N*-terminal structure and in particular the *N*-glycosylation of GPCRs may influence a range of receptor functions, including the trafficking of receptors to the cell surface, ligand binding, receptor activation and down-regulation [39].

SNPs within *HTR4* have been associated with lung function [5,6] and COPD/airway obstruction [8,9] in several populations. Recently, *HTR4* SNPs have also been associated with asthma in a Korean population, with the most significant associations localized to introns within *HTR4* that were previously identified in the SpiroMeta/CHARGE lung function meta-analyses [5,6,40]. However, large scale analyses of asthma utilizing 10,365 asthma and 16,110 unaffected controls as part of the GABRIEL study did not identify an association for *HTR4* SNPs [41].

The functional relevance of associated *HTR4* SNPs remains to be determined, however the potential localization of the key SNPs (*i.e.* rs3995090 and rs6889822) from the SpiroMeta lung function meta-analyses to intron 6 of the gene (variant a) provided an initial focus for the current study. The ENCODE database identified a number of features within this locus (histone modifications, DNase I hypersensitivity clusters and transcription factor binding sites) consistent with a regulatory region. Interestingly, according to the HaploReg database, three of the SNPs (rs7733088, rs4705259 and 5:147836450) in LD (r^2^>0.80) with the sentinel SNP (rs3995090) identified in the SpiroMeta lung function GWAS significantly alter Foxp1 binding motifs within *HTR4*. The Fox family of transcription factors are key regulators of lung development. In particular, the sub-family of Foxp1/2/4 is highly expressed in the developing airway epithelium, as well as in the adult lung [42]. Foxp1 and Foxp2 co-operatively regulate distal lung epithelial development [43], while Foxp1 and Foxp4 together modulate lung secretory epithelial cell fate during development and regeneration, by restricting the goblet cell differentiation program [44]. Given the presence of a Foxp1 binding motif within the region of *HTR4* associated with lung function, it is tempting to speculate that Foxp1 binding in this locus might be important in regulating *HTR4* during lung development.

Influences on airway growth during development can have long-term physiological effects within the lung [45]. Our data suggest that *HTR4* is differentially expressed in the developing lung. When taken together with the finding that SNPs in LD with those associated with lung function alter transcription factor binding sites for factors known to be involved in lung development, our results suggest that *HTR4* expression may be important for lung development. In agreement with this interpretation, it is important to note that the association between *HTR4* SNPs and lung function was apparent in children included in GWAS meta-analyses of lung function, *e.g.* in the ALSPAC (Avon Longitudinal Study of Parents and Children) and in the Raine Studies [6,7].

It is also important to note that a role for 5-HT_4_R in post-natal lung related functions has been described including: the modulation of cytokine release in human alveolar type II cells [16], influencing the effect of 5-HT on cholinergic contraction in human bronchial strips [46] and bronchopulmonary C-fibre mediated cough and dyspnea [47]. Similarly, 5-HT_4_R is expressed in the pre-Bötzinger complex in the CNS, and has been demonstrated to regulate spontaneous respiratory activity [48].

## Conclusions

In summary, we have demonstrated that *HTR4* is expressed at the mRNA and protein level in adult lung tissue, albeit at a low level, using both normal and COPD lung tissue sections and cultured airway cells. The analyses of lung tissue from different fetal stages of development highlight differential expression of *HTR4* at the protein and mRNA level, suggesting a developmental role. Intriguingly, we have also identified that the key SNPs in *HTR4* associated with lung function also potentially alter binding of a series of key transcription factors of relevance for the development of multiple organs. Finally, we further characterized the gene structure of *HTR4*, identifying in brain a novel splice variant with potential functional implications. These data represent a significant advance in our knowledge of the role of *HTR4* in the lung and provide the first steps towards the translation of population-based genetic association into respiratory biology.

## Abbreviations

5HT: 5-hydroxytryptamine; 5-HT4R: 5-HT receptor type 4; COPD: Chronic obstructive pulmonary disease; ENCODE: Encyclopedia of DNA elements consortium; FEV1: Forced expiratory volume in 1 s; FVC: Forced vital capacity; GPCR: G protein-coupled receptor; GWAS: Genome-wide association study; HASM: Human airway smooth muscle; HBEC: Human bronchial epithelial cells; HTR4: Gene encoding 5-HT receptor type 4; IHC: Immunohistochemistry; LD: Linkage disequilibrium; PCR: Polymerase chain reaction; Q-PCR: Quantitative PCR; RACE: Rapid amplification of cDNA ends; RT-PCR: Reverse transcription PCR; SNP: Single nucleotide polymorphism.

## Competing interests

The authors have no competing interests to declare.

## Authors’ contributions

IS, IPH and AM conceived and designed the study. EH/CN/IS drafted the manuscript. EH, CES, CKB, AH and CS performed the RT-PCR, while CG and CS completed the Q-PCR. AH, CKB and CS performed the RACE studies. SM completed the IHC. CN performed the radioligand binding experiments. CN and AH performed the bioinformatic analysis. EM collaborated on the Affymetrix data analyses. All authors contributed to the final draft of the manuscript.

## Supplementary Material

Additional file 1: Table S1 Predicted regulatory effects of key *HTR4* SNPs. Click here for file

## References

[B1] Berger M Gray JA Roth BL The expanded biology of serotonin Annu Rev Med 2009 60 355 366 10.1146/annurev.med.60.042307.110802 19630576 PMC5864293

[B2] Kroeze WK Kristiansen K Roth BL Molecular biology of serotonin receptors structure and function at the molecular level Curr Top Med Chem 2002 2 507 528 12052191 10.2174/1568026023393796

[B3] Hoyer D Hannon JP Martin GR Molecular, pharmacological and functional diversity of 5-HT receptors Pharmacol Biochem Behav 2002 71 533 554 10.1016/S0091-3057(01)00746-8 11888546

[B4] Milligan G G protein-coupled receptor hetero-dimerization: contribution to pharmacology and function Br J Pharmacol 2009 158 5 14 10.1111/j.1476-5381.2009.00169.x 19309353 PMC2795239

[B5] Hancock DB Eijgelsheim M Wilk JB Gharib SA Loehr LR Marciante KD Franceschini N van Durme YM Chen TH Barr RG Meta-analyses of genome-wide association studies identify multiple loci associated with pulmonary function Nat Genet 2010 42 45 52 10.1038/ng.500 20010835 PMC2832852

[B6] Repapi E Sayers I Wain LV Burton PR Johnson T Obeidat M Zhao JH Ramasamy A Zhai G Vitart V Genome-wide association study identifies five loci associated with lung function Nat Genet 2010 42 36 44 10.1038/ng.501 20010834 PMC2862965

[B7] Soler Artigas M Loth DW Wain LV Gharib SA Obeidat M Tang W Zhai G Zhao JH Smith AV Huffman JE Genome-wide association and large-scale follow up identifies 16 new loci influencing lung function Nat Genet 2011 43 1082 1090 10.1038/ng.941 21946350 PMC3267376

[B8] Soler Artigas M Wain LV Repapi E Obeidat M Sayers I Burton PR Johnson T Zhao JH Albrecht E Dominiczak AF Effect of five genetic variants associated with lung function on the risk of chronic obstructive lung disease, and their joint effects on lung function Am J Respir Crit Care Med 2011 184 786 795 10.1164/rccm.201102-0192OC 21965014 PMC3398416

[B9] Wilk JB Shrine NR Loehr LR Zhao JH Manichaikul A Lopez LM Smith AV Heckbert SR Smolonska J Tang W Genome-wide association studies identify CHRNA5/3 and HTR4 in the development of airflow obstruction Am J Respir Crit Care Med 2012 186 622 632 10.1164/rccm.201202-0366OC 22837378 PMC3480517

[B10] Coupar IM Desmond PV Irving HR Human 5-HT(4) and 5-HT(7) receptor splice variants: are they important? Curr Neuropharmacol 2007 5 224 231 10.2174/157015907782793621 19305739 PMC2644495

[B11] Bender E Pindon A van Oers I Zhang YB Gommeren W Verhasselt P Jurzak M Leysen J Luyten W Structure of the human serotonin 5-HT4 receptor gene and cloning of a novel 5-HT4 splice variant J Neurochem 2000 74 478 489 10646498 10.1046/j.1471-4159.2000.740478.x

[B12] Bockaert J Claeysen S Compan V Dumuis A 5-HT(4) receptors: history, molecular pharmacology and brain functions Neuropharmacology 2008 55 922 931 10.1016/j.neuropharm.2008.05.013 18603269

[B13] Hegde SS Eglen RM Peripheral 5-HT4 receptors FASEB J 1996 10 1398 1407 8903510 10.1096/fasebj.10.12.8903510

[B14] Bach T Syversveen T Kvingedal AM Krobert KA Brattelid T Kaumann AJ Levy FO 5HT4(a) and 5-HT4(b) receptors have nearly identical pharmacology and are both expressed in human atrium and ventricle Naunyn Schmiedebergs Arch Pharmacol 2001 363 146 160 10.1007/s002100000299 11218067

[B15] Brattelid T Kvingedal AM Krobert KA Andressen KW Bach T Hystad ME Kaumann AJ Levy FO Cloning, pharmacological characterisation and tissue distribution of a novel 5-HT4 receptor splice variant, 5-HT4(i) Naunyn Schmiedebergs Arch Pharmacol 2004 369 616 628 10.1007/s00210-004-0919-4 15118808

[B16] Bayer H Muller T Myrtek D Sorichter S Ziegenhagen M Norgauer J Zissel G Idzko M Serotoninergic receptors on human airway epithelial cells Am J Respir Cell Mol Biol 2007 36 85 93 10.1165/rcmb.2006-0151OC 16873768

[B17] Einstein R Jordan H Zhou W Brenner M Moses EG Liggett SB Alternative splicing of the G protein-coupled receptor superfamily in human airway smooth muscle diversifies the complement of receptors Proc Natl Acad Sci USA 2008 105 5230 5235 10.1073/pnas.0801319105 18362331 PMC2278184

[B18] Liu B Peel SE Fox J Hall IP Reverse mode Na+/Ca2+ exchange mediated by STIM1 contributes to Ca2+ influx in airway smooth muscle following agonist stimulation Respir Res 2010 11 168 10.1186/1465-9921-11-168 21126331 PMC3012663

[B19] Stewart CE Nijmeh HS Brightling CE Sayers I uPAR regulates bronchial epithelial repair in vitro and is elevated in asthmatic epithelium Thorax 2012 67 477 487 10.1136/thoraxjnl-2011-200508 22139533 PMC3358731

[B20] Reddel RR Ke Y Kaighn ME Malan-Shibley L Lechner JF Rhim JS Harris CC Human bronchial epithelial cells neoplastically transformed by v-Ki-ras: altered response to inducers of terminal squamous differentiation Oncogene Res 1988 3 401 408 3067190

[B21] Livak KJ Schmittgen TD Analysis of relative gene expression data using real-time quantitative PCR and the 2(−Delta Delta C(T)) Method Methods 2001 25 402 408 10.1006/meth.2001.1262 11846609

[B22] Melen E Kho AT Sharma S Gaedigk R Leeder JS Mariani TJ Carey VJ Weiss ST Tantisira KG Expression analysis of asthma candidate genes during human and murine lung development Respir Res 2011 12 86 10.1186/1465-9921-12-86 21699702 PMC3141421

[B23] Kho AT Bhattacharya S Tantisira KG Carey VJ Gaedigk R Leeder JS Kohane IS Weiss ST Mariani TJ Transcriptomic analysis of human lung development Am J Respir Crit Care Med 2010 181 54 63 10.1164/rccm.200907-1063OC 19815808 PMC2797628

[B24] Ponimaskin E Dumuis A Gaven F Barthet G Heine M Glebov K Richter DW Oppermann M Palmitoylation of the 5-hydroxytryptamine4a receptor regulates receptor phosphorylation, desensitization, and beta-arrestin-mediated endocytosis Mol Pharmacol 2005 67 1434 1443 10.1124/mol.104.008748 15689570

[B25] Krogh A Larsson B von Heijne G Sonnhammer EL Predicting transmembrane protein topology with a hidden Markov model: application to complete genomes J Mol Biol 2001 305 567 580 10.1006/jmbi.2000.4315 11152613

[B26] Rosenbloom KR Dreszer TR Long JC Malladi VS Sloan CA Raney BJ Cline MS Karolchik D Barber GP Clawson H ENCODE whole-genome data in the UCSC Genome Browser: update 2012 Nucleic Acids Res 2012 40 D912 D917 10.1093/nar/gkr1012 22075998 PMC3245183

[B27] Kent WJ Sugnet CW Furey TS Roskin KM Pringle TH Zahler AM Haussler D The human genome browser at UCSC Genome Res 2002 12 996 1006 12045153 10.1101/gr.229102 PMC186604

[B28] Meyer LR Zweig AS Hinrichs AS Karolchik D Kuhn RM Wong M Sloan CA Rosenbloom KR Roe G Rhead B The UCSC Genome Browser database: extensions and updates 2013 Nucleic Acids Res 2013 41 D64 D69 10.1093/nar/gks1048 23155063 PMC3531082

[B29] Ward LD Kellis M HaploReg: a resource for exploring chromatin states, conservation, and regulatory motif alterations within sets of genetically linked variants Nucleic Acids Res 2012 40 D930 D934 10.1093/nar/gkr917 22064851 PMC3245002

[B30] Blondel O Vandecasteele G Gastineau M Leclerc S Dahmoune Y Langlois M Fischmeister R Molecular and functional characterization of a 5-HT4 receptor cloned from human atrium FEBS Lett 1997 412 465 474 10.1016/S0014-5793(97)00820-X 9276448

[B31] Claeysen S Sebben M Becamel C Bockaert J Dumuis A Novel brain-specific 5-HT4 receptor splice variants show marked constitutive activity: role of the C-terminal intracellular domain Mol Pharmacol 1999 55 910 920 10220570

[B32] Vilaro MT Domenech T Palacios JM Mengod G Cloning and characterization of a novel human 5–HT4 receptor variant that lacks the alternatively spliced carboxy terminal exon. RT-PCR distribution in human brain and periphery of multiple 5–HT4 receptor variants Neuropharmacology 2002 42 60 73 10.1016/S0028-3908(01)00154-X 11750916

[B33] Medhurst AD Lezoualc’h F Fischmeister R Middlemiss DN Sanger GJ Quantitative mRNA analysis of five C-terminal splice variants of the human 5-HT4 receptor in the central nervous system by TaqMan real time RT-PCR Brain Res Mol Brain Res 2001 90 125 134 10.1016/S0169-328X(01)00095-X 11406291

[B34] Bonaventure P Hall H Gommeren W Cras P Langlois X Jurzak M Leysen JE Mapping of serotonin 5-HT(4) receptor mRNA and ligand binding sites in the post-mortem human brain Synapse 2000 36 35 46 10.1002/(SICI)1098-2396(200004)36:1<35::AID-SYN4>3.0.CO;2-Y 10700024

[B35] Tadevosyan A Vaniotis G Allen BG Hebert TE Nattel S G protein-coupled receptor signalling in the cardiac nuclear membrane: evidence and possible roles in physiological and pathophysiological function J Physiol 2012 590 1313 1330 22183719 10.1113/jphysiol.2011.222794 PMC3382322

[B36] Wai MS Lorke DE Kwong WH Zhang L Yew DT Profiles of serotonin receptors in the developing human thalamus Psychiatry Res 2011 185 238 242 10.1016/j.psychres.2010.05.003 20538346

[B37] Lambe EK Fillman SG Webster MJ Shannon Weickert C Serotonin receptor expression in human prefrontal cortex: balancing excitation and inhibition across postnatal development PLoS One 2011 6 e22799 10.1371/journal.pone.0022799 21829518 PMC3146513

[B38] Azim S Banday AR Tabish M Identification of alternatively spliced multiple transcripts of 5-hydroxytryptamine receptor in mouse Brain Res Bull 2012 87 250 258 10.1016/j.brainresbull.2011.10.016 22079627

[B39] Unal H Karnik SS Domain coupling in GPCRs: the engine for induced conformational changes Trends Pharmacol Sci 2012 33 79 88 10.1016/j.tips.2011.09.007 22037017 PMC3273637

[B40] Kim TH An SH Cha JY Shin EK Lee JY Yoon SH Lee YM Uh ST Park SW Park JS Association of 5-hydroxytryptamine (serotonin) receptor 4 (5-HTR4) gene polymorphisms with asthma Respirology 2011 16 630 638 10.1111/j.1440-1843.2011.01963.x 21382128

[B41] Moffatt MF Gut IG Demenais F Strachan DP Bouzigon E Heath S von Mutius E Farrall M Lathrop M Cookson WO A large-scale, consortium-based genomewide association study of asthma N Engl J Med 2010 363 1211 1221 10.1056/NEJMoa0906312 20860503 PMC4260321

[B42] Lu MM Li S Yang H Morrisey EE Foxp4: a novel member of the Foxp subfamily of winged-helix genes co-expressed with Foxp1 and Foxp2 in pulmonary and gut tissues Mech Dev 2002 119 Suppl 1 S197 S202 14516685 10.1016/s0925-4773(03)00116-3

[B43] Shu W Lu MM Zhang Y Tucker PW Zhou D Morrisey EE Foxp2 and Foxp1 cooperatively regulate lung and esophagus development Development 2007 134 1991 2000 10.1242/dev.02846 17428829

[B44] Li S Wang Y Zhang Y Lu MM DeMayo FJ Dekker JD Tucker PW Morrisey EE Foxp1/4 control epithelial cell fate during lung development and regeneration through regulation of anterior gradient 2 Development 2012 139 2500 2509 10.1242/dev.079699 22675208 PMC3383227

[B45] Stick S Pediatric origins of adult lung disease. 1. The contribution of airway development to paediatric and adult lung disease Thorax 2000 55 587 594 10.1136/thorax.55.7.587 10856320 PMC1745803

[B46] Dupont LJ Pype JL Demedts MG De Leyn P Deneffe G Verleden GM The effects of 5-HT on cholinergic contraction in human airways in vitro Eur Respir J 1999 14 642 649 10.1034/j.1399-3003.1999.14c26.x 10543288

[B47] Potenzieri C Meeker S Undem BJ Activation of mouse bronchopulmonary C-fibres by serotonin and allergen-ovalbumin challenge J Physiol 2012 590 5449 5459 10.1113/jphysiol.2012.237115 22907059 PMC3515830

[B48] Manzke T Guenther U Ponimaskin EG Haller M Dutschmann M Schwarzacher S Richter DW 5-HT4(a) receptors avert opioid-induced breathing depression without loss of analgesia Science 2003 301 226 229 10.1126/science.1084674 12855812

