# Supplementary material for: HTR4 gene structure and altered expression in the developing lung
Source: Respir Res. 2013 Jul 26;14(1):77. doi: 10.1186/1465-9921-14-77 (PMC3750317; doi:10.1186/1465-9921-14-77)
Supplement: Additional file 1: Table S1 — Predicted regulatory effects of key HTR4 SNPs. [file 1465-9921-14-77-S1.pdf]

Additional Data File

HTR4 gene structure and altered expression in the developing lung

*Emily Hodge[1], *Carl P Nelson[1], *Suzanne Miller[1], Charlotte K Billington[1], Ceri E Stewart[1], Caroline Swan[1], Anders Malarstig[3], Amanda P Henry[1], Catherine Gowland[1], Erik Melén[2], Ian P Hall[1] and Ian Sayers[1]

[1]Division of Therapeutics and Molecular Medicine, University of Nottingham, Nottingham, United Kingdom

[2]Institute of Environmental Medicine, Karolinska Institutet and Sachs' Children's Hospital, Stockholm, Sweden

[3] Precision Medicine Unit, Pfizer Global Research and Development, Cambridge, United Kingdom

*contributed equally

Correspondence should be addressed to:

Dr Ian Sayers,

Division of Therapeutics and Molecular Medicine,

University of Nottingham,

Queen's Medical Centre,

Nottingham NG7 2UH, UK.

Tel.: +44(0)115 8231066

Fax: +44(0)115 8231059

E-mail: Ian.Sayers@nottingham.ac.uk.

This study was funded by grants from the Medical Research Council, UK (G1000861) and Pfizer Inc.

Supplementary Table 1.

Position	Variant	LD	Enhancer histone marks	DNase hyper-sensitivity	Proteins bound	Motifs altered
147836609	rs13156542	0.93	No	-	-	-
147836880	rs12374521	0.93	No	**HBMEC**<br>**HFF-Myc**	**CTCF**	*Nkx2*
147838844	rs10463406	1	No	-	-	-
147839537	rs10075211	0.93	No	**HCF**	-	*RXR::LXR*<br>*RAR*<br>*PPARG_1*<br>*HNF4_6*<br>*Err-alpha*
147840112	rs10041517	0.96	No	-	-	-
147840828	rs6860087	0.96	No	-	-	*Mef2*
147840830	rs6860089	0.96	No	**-**	-	*Mef2*
147842353	rs11168048	0.93	No	-	-	-
147843267	rs4705259	0.96	No	-	-	*Foxd3*<br>*Foxi1*<br>*Fox*<br>*Foxj2_1*<br>*Foxa_3*<br>*Foxp1*<br>*Foxa_1*<br>*Pax-4_4*<br>*Foxj_1*
147844392	rs7735184	1	No	-	-	-
147845815	**rs3995090**	1	No	**PANC-1**	-	*Obox3*
147846403	rs11742110	1	No	-	-	-
147846707	**rs6889822**	0.96	No	-	-	-
147847788	rs1985524	0.93	No	-	-	*NRSF*
147848646	rs1989153	0.93	**Yes***	-	-	-
147848890	rs1989154	0.93	**Yes***	**HPF**	-	-
147849531	rs1988819	0.93	**Yes***	-	-	-
147849635	rs1988818	0.93	**Yes***	-	-	-
147849759	rs3995091	0.93	**Yes***	-	-	*AFP1*<br>*Hoxb8*<br>*Mef2*
147851270	rs6887366	0.93	**Yes***	**AG04449**	**CFOS**	-
147852612	rs7736804	0.96	No	-	-	-
147854608	rs60611116	0.96	No	-	-	-
147854970	rs10037493	0.90	No	-	-	-
147855264	rs11168049	0.96	No	-	-	-
147856232	rs6580550	0.86	No	-	-	-
147856257	5:147836450	0.93	No	-	-	*Foxp1*
147856333	rs7733088	0.96	No	-	-	*Hoxc6*<br>*Pou4f3*<br>*Pou2f1_10*<br>*Foxp1*<br>*Nkx6-1_2*<br>*Pou3f4*<br>*Prrx1*<br>*Pou6f1_1*<br>*Hoxa7_2*

						Hlx1<br>*Nkx6-1_3*
147856392	rs7715901	0.96	No	-	-	*Foxa*
147856522	rs7733410	0.86	No	-	-	*EWSR1-FLI1*<br>*NR3C*

* strong enhancer in HUVEC cells

Supplementary Table 1. Predicted regulatory effects of key *HTR4* SNPs. *HTR4* variants in LD (r^2>0.80) with the sentinel SNP (rs3995090) identified in the SpiroMeta lung function GWAS (11), their genomic positions (on the Human Feb 2009 (GRCh37/hg19) assembly) and their predicted effects on DNA regulatory elements. Data were obtained using the HaploReg database, hosted by The Broad Institute (12). The top two SNPs identified within *HTR4* in the SpiroMeta GWAS (rs3995090 and rs6889822) are indicated in bold. HBMEC = human brain microvascular endothelial cells; HFF-Myc = human foreskin fibroblasts expressing the cMyc gene; HCF = human cardiac fibroblasts; PANC-1 = human pancreatic carcinoma, epithelial-like cells; HPF = human pulmonary fibroblasts; AG04449 = fetal buttock/thigh fibroblasts; HUVEC = human umbilical vein endothelial cells.
